# Outcomes of Transferred Adult Venovenous and Venoarterial Extracorporeal Membrane Oxygenation Patients: A Single Center Experience

**DOI:** 10.3389/fmed.2022.913816

**Published:** 2022-06-13

**Authors:** Yang-Chao Zhao, Xi Zhao, Guo-Wei Fu, Ming-Jun Huang, Hui Zhao, Zhen-Qing Wang, Xing-Xing Li, Jun Li

**Affiliations:** ^1^Department of Extracorporeal Life Support Center, Department of Cardiac Surgery, The First Affiliated Hospital of Zhengzhou University, Zhengzhou, China; ^2^Department of Cardiology, Cardiovascular Center, Henan Key Laboratory of Hereditary Cardiovascular Diseases, The First Affiliated Hospital of Zhengzhou University, Zhengzhou, China

**Keywords:** transportation, extracorporeal membrane oxygenation, intensive care unit mortality, mean arterial pressure, complications

## Abstract

**Objectives:**

Extracorporeal membrane oxygenation (ECMO) patients with or without transport both have high hospital mortality rate and there are few data on adult VA-ECMO transport patients. Hence, this study was designed to analyze factors that affect the outcomes of patients with ECMO transport.

**Methods:**

This study retrospectively enrolled 126 ECMO patients transferred from regional hospital to the First Affiliated Hospital of Zhengzhou University by our ECMO team during June 2012 to Sept 2020. Data were calculated and analyzed.

**Results:**

The median distance of transportation was 141 (76–228) km, the median transport time consuming was 3 (1.3–4) h, the percentage of complications during transport was 40.5% (except for bleeding on cannula site, and no one death during transport), and the survival rate in hospital was 38.9%. Compared with survivors, the non-survivors were older and showed higher SOFA score, longer time with ECMO assisted, longer time in ICU and in hospital. However, after divided into VA-ECMO and VV-ECMO groups, the older age showed no significant difference between survivors and non-survivors groups of VA-ECMO patients. Moreover, the Cox regression survival analysis showed that higher SOFA score and lactate level indicated higher ICU mortality of VA-ECMO patients while higher SOFA score, higher lactate level, older age and lower MAP after transportation (<70mmHg) indicated higher ICU mortality of VV-ECMO patients. However, there was no significant difference of comorbidities and complications in survivors and non-survivors groups of ECMO patients.

**Conclusions:**

The transportation for ECMO patients can be feasible performed although life-threatening complications might occur. The SOFA score and the lactate level could be used to evaluate the risk of ICU mortality of transportation ECMO patients. Besides, lower MAP after transportation (<70mmHg) had potential predictive value for short-term outcome of VV-ECMO patients.

## Introduction

For patients with severe reversible refractory respiratory or circulatory failure, extracorporeal membrane oxygenation (ECMO) is now recognized as a lifesaving procedure. The Extracorporeal Life Support Organization (ELSO), an authoritative organization, collects a large amount of ECMO-related data and provides therapy guidelines including ECMO transportation programs. The mortality of specialized high-volume centers was showed lower than that of regional hospitals and the ELSO recommends transported ECMO-supported individuals to centers at least 30 adult supported individuals per year (1). Other large-scale studies have also confirmed that interhospital ECMO transports to large-volume ECMO centers reduces mortality significantly (2–4).

The survival rate of ECMO supported patients was low. A referral center compared the outcomes of 51 transferred and 215 in-house venoarterial ECMO (VA-ECMO) supported patients and found there was no significant difference in the mortality rate (56.7 vs. 60.8%) (5). The mortality rate of ECMO-supported patients during transportation was reported as low as 0.15% (6–9). It seems that initiating ECMO at an outlying hospital and transferring patients to large referral center for continued care may result in similar survival outcomes. However, the transferred and in-house ECMO supported patients existed many differences. First of all, the composition of the two types of patients is different. There are fewer adults and VA models for transporting patients, and patients who are overly ill may not choose to be transported. Secondly, the medical conditions of the transfer vehicle are not as good as in the hospital, and it also involves the cooperation of the ECMO team, the occurrence of complications during the transfer, and the maintenance of various indicators during the transportation. Therefore, although the prognosis of the two types of patients is similar, we hypothesized that there may be different risk factors affecting the transportation ECMO patients' prognosis. In the present study, we enrolled 126 adult transferred ECMO patients. Among them, the proportion of VA-ECMO was larger than that of previous studies. We calculated their baseline characteristics and indicators at three different time points, and then analyzed the impact of different indicators on short-term prognosis.

The aim of this work is to seek factors that may affect and even could evaluate the survival rate of VA and venovenous (VV) ECMO supported transportation patients.

## Materials and Methods

### Study Design and Patients

The present study fully complied with the Declaration of Helsinki and was approved by the Ethics Committee of the First Affiliated Hospital of Zhengzhou University, Zhengzhou, China (no. 2020-KY-429).

**Figure 1:**
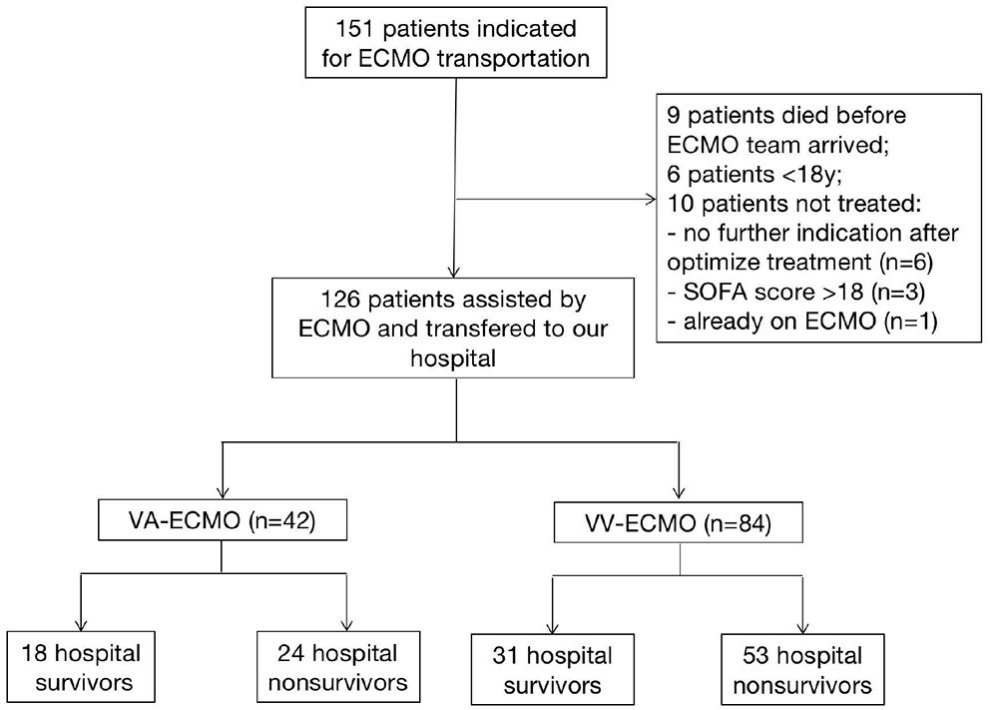
The flow chart.

Our study retrospectively enrolled adult patients treated with ECMO in regional hospitals by our mobile teams and transferred to the First Affiliated Hospital of Zhengzhou University immediately from June 2012 to Sept 2020. The exclusion criteria were as follows: 1) aged <18 years old; 2) the sequential organ failure assessment (SOFA) score >18; 3) low platelet count (<50 × 10^12^/L), intracranial hemorrhage or other contraindications to heparin treatment; 4) no further indication after optimal treatment; and 4) secondary transports (the patients is already on ECMO before our team arrived) (Figure 1). Finally, 42 VA-ECMO supported patients and 84 VV-ECMO ones who had indications for ECMO transportation were enrolled and then divided into hospital survival group and non-survival group.

The clinical data, modes of support (venovenous or venoarterial), the time of ECMO assisted, mechanical ventilation, intensive care unit (ICU) stay and hospital stay were collected. The indications for ECMO, the distance, the time consuming and the complications of transportation were recorded. Complications of transported ECMO patients were divided into equipment complications and patients ones (2). Equipment complications were consisted by clotting of ECMO system, pump failure, ECMO system air embolism and oxygen deficit (9). Clotting of ECMO system was that a blood clot larger than 3.0 mm can be seen near the membrane. Pump failure was referred to the sudden stop of the centrifugal pump. ECMO system air embolism was defined as more than 30 ml of air entering the ECMO system, which cannot be discharged by itself, and needs to be manually exhausted. Oxygen deficit was the large amount of oxygen used by ventilators and ECMO machines in transferred patients and needs to be deployed urgently. The patient complications were containing hemorrhage (respiratory and/or gastrointestinal tract hemorrhage, intracranial hemorrhage and bleeding on cannula site), lower limb ischemia, emergency orotracheal intubation, cardiac arrest, scald and epilepsy. According to the ELSO definition of severe bleeding (10), hemorrhagic complication was defined as: clinically significant bleeding requiring the administration of 2 packed red blood cells or more within 24 h, or a drop in hemoglobin of at least 2 g/dL within 24 h excluding haemolysis and/or bleeding from specific sites such as the central nervous system and/or bleeding requiring specific interventions such as embolisation, surgery, etc. Lower limb ischemia was referred to ischemia manifestations such as coldness, mottled, cyanosis, etc. on either side of the lower limbs. Emergency orotracheal intubation was a sharp drop in the oxygenation index which need to orotracheal intubation immediately during the transfer of ECMO patients. Cardiac arrest was defined as patient's heart stops beating during transit and CPR is required. Scald was the formation of skin blisters caused by the main pump of EMCO contacting the body of the patient and the main pump being heated for a long time. Seizures was epileptic symptoms such as convulsions during transport. The severity of the illness was assessed based on the SOFA score before ECMO initiation. Besides, heart rate (HR), respiratory rate (R), mean arterial pressure (MAP), the levels of lactate, the arterial partial pressure of oxygen (PaO_2_) and the arterial partial pressure of carbon dioxide (PaCO_2_) were obtained in three time points (before ECMO boarding, after ECMO boarding and patient's condition is relatively stable, transported to the hospital).

### Mobile ECMO Team and Equipment

The composition of the mobile ECMO team currently has no clear guidelines but has recommendations. A mature ECMO team usually including physicians, transport specialists, nurses, perfusionists, and other ECMO specialists (9). According to the recommendations, our mobile ECMO team consists of ECMO physician, emergency physician or intensive care physician, and intensive care nurse. Firstly, the team will evaluate the patient and indication of ECMO. Once transportation is needed, ECMO physician should manage the cannulation. The mobile ECMO team was in charge of the ECMO circuit, the ventilator, medications, the application of heparin, and resolve complications of transferred critically ill patient. Besides, the cannulation is often performed through percutaneously by ECMO physician.

All transport-related coordination was handled by the ECMO physician. The ECMO team, equipment, and critically ill patients were ground transferred by emergency ambulance. ECMO system used were ROTAFLOW centrifugal pump (Maquet Cardiopulmonary, Rastatt, Germany), SCP/SCPC (Stöckert, Munchen, Germany) or Bio-Console 560 (Medtronic,Minneapolis, USA). The ECMO oxygenator was MEDOS HILITE 7000LT (Medos Medizintechnik, Stolberg, Germany) or D905 EOS ECMO(Sorin, Mirandola, ITALY). All cannulations were peripheral. There were two approaches of cannulation in the transferred patients as femoro-femoral cannulation approach used in VA-ECMO assisted patients while femoro-jugular in VV-ECMO ones. For VV/VA ECMO, the following single lumen cannulas were used: Bio-Medicus 15–21 French (Fr)/18 cm, 15–21 Fr/50 cm (Medtronic); BE-PAL 15–21 Fr/23 cm,BE-PVL 19–23 Fr/55 cm (Maquet); OPTI 16–22 Fr/24cm, VFEM 18–22 Fr/55 cm(Edwards); or BMA 16–26 Fr/38.3 cm,BMA 18–28 Fr/80.7 cm(Medos). During transport, an MAQUET SERVO-i ventilator was used for patient monitoring, while blood gases and activated clotting times were assessed by using an MD-125(Beijing, China), MINI-II(Beaumont, Texas, USA) or Bio Trend(Medtronic, Minneapolis, USA).

### Statistical Analysis

All collected data were statistically analyzed using SPSS 21.0 (Armonk, NY: IBM Corp.). Measurement data are presented as mean (± SD) or median (IQR), and the two groups compared by analysis of variance. Count data were expressed by frequency (composition ratio), and comparison between groups was by χ2 test or Fisher's exact test. *P* < 0.05 indicates that the difference is statistically significant. Survivor /non-survivor was selected for the dependent variable for the Cox regression analysis. Based on comparison of baseline information and relevant indicators, the independent variable for VA-ECMO patients was selected as age, SOFA score, post-transfer MAP and baseline lactate level, while for VV-ECMO patients as age, SOFA score, baseline lactate level, post-transfer heart rate, post-transfer respiratory rate and post-transfer MAP. The ROC curve was used to analyze the potential predictor for ICU mortality of ECMO patients and to find the cut-off value. The Cox regression survival analysis was performed to searching the risk factors of poor prognosis.

## Results

### Characteristics of Transferred ECMO Patients

The characteristics of 126 enrolled patients are shown in Tables 1, 2. The median distance of transportation was 141 (76–228) km, and the median transport time consuming was 3 (1.3–4) h (Table 1). As shown in Table 1, the transferred patients with 46.8% being male and the mean age was 45 ± 14 years old were divided into survival and non-survival group. Of the two groups, there was no significant difference in patients' gender, BMI, comorbidities, cannulation time consuming, distance of transportation, transport time consuming, ventilation time, remote shunt, the application of auxiliary equipment (intra-aortic balloon pump/IABP and continuous renal replacement therapy/CRRT), and the percentage of VA/VV ECMO (*p* > 0.05, Table 1). Compared with survivors, the non-survivors were older and showed higher SOFA score, longer time with ECMO assisted, longer time in ICU and in hospital (age: 49 ± 13 years vs.39 ± 13 years; median SOFA score: 11(10–12) vs. 8(6.5–11); median ECMO assisted time: 256(129–372.5) h vs. 176 (121– 204.5) h; time in ICU:13.3 ± 5.9 days vs. 21.4 ± 6.2days; time in hospital: 14.8 (10.1–17.5) days vs. 22 (17.8–32.3) days; all *P* < 0.001, respectively, Table 1). However, after divided into VA-ECMO and VV-ECMO groups, the older age showed no significant difference between survivors and non-survivors groups of VA-ECMO patients (*p* = 0.132) but remain significantly difference in VV-ECMO group (39 ± 12 years vs. 51 ± 13 years, *P* < 0.001, Table 2). Furthermore, the differences of SOFA score were still exist in survivors and non-survivors groups of VA and VV ECMO patients (VA-ECMO group: 10 (8–11) vs. 12 (10.25–12.75), *p* = 0.003; VV-ECMO group: 8 (6–11) vs. 11 (9.5-12), *P* < 0.001; respectively, Table 3).

**Table 1:** Baseline characteristics of transferred ECMO patients.

	**Total** **(*n =* 126)**	**Non–survival Group** **(*n =* 77)**	**Survival Group** **(*n =* 49)**	***P*–value**
Male gender, (*n*%)	59 (46.8)	37 (48.1)	22 (44.9)	0.732
Age (y)	45 ± 14	49 ± 13	39 ± 13	**<0.001**
BMI (Kg/M2)	23.6 ± 3.4	23.6 ± 3.0	23.7 ± 4.0	0.898
SOFA score	10 (8–12)	11 (10–12)	8 (6.5–11)	**<0.001**
Comorbidities, (*n*%)				
Type 2 diabetes	27 (21.4)	15 (19.5)	12 (24.5)	0.215
Hypertension	35 (27.8)	20 (25.9)	15 (30.6)	0.382
COPD	23 (18.3)	13 (16.9)	10 (20.4)	0.522
Indications for ECMO, (*n*%)				0.369
Pneumonia	55 (43.7)	34 (44.2)	21 (42.9)	
Fulminant myocarditis	11 (8.7)	7 (9.1)	4 (8.2)	
Coronary heart disease	9 (7.1)	3 (3.9)	6 (12.2)	
Others	51 (40.5)	33 (42.9)	18 (36.7)	
Cannulation time consuming (min)	31.8 ± 10.8	31.0 ± 10.8	33.2 ± 10.7	0.266
Distance of transportation (km)	141 (76–228)	141 (77–228)	141 (39.5–228)	0.459
Transport time consuming (h)	3 (1.3–4)	3 (1.4–3.9)	3 (1.1–4.5)	0.779
Liquid per hours (ml/h)	76 (41–138)	80 (45–155)	67 (35–116)	0.319
Urine per hours (ml/h)	45 (25–83)	45 (20–82)	44 (29–99)	0.189
Remote shunt (*n* %)	10 (7.9)	3 (3.9)	7 (14.3)	0.064
IABP (*n* %)	26 (20.6)	14 (18.2)	12 (24.5)	0.593
CRRT (*n* %)	82 (65.1)	48 (62.3)	34 (69.4)	0.422
Ventilation time, (h)	298.8 ± 142.5	306.9 ± 136.0	284.2 ± 154.1	0.411
ECMO assisted time, (h)	196 (124.5–324.8)	256 (129–372.5)	176 (121–204.5)	**<0.001**
Time in ICU, (d)	16.4 ± 7.2	13.3 ± 5.9	21.4 ± 6.2	**<0.001**
Time in hospital, (d)	17 (12.8–21.2)	14.8 (10.1–17.5)	22 (17.8–32.3)	**<0.001**
VA/VV ECMO, (*n*%)	42 (33.3)	24 (31.2)	18 (36.7)	0.522

*The data was shown as the mean ± SD, median (interquartile 25–75) or n (percentage). Bold values indicate statistical significance. BMI, body mass index; COPD, chronic obstructive pulmonary disease; SOFA, sepsis–related organ failure assessment; IABP, intra–aortic balloon pump; CRRT, continuous renal replacement therapy; ICU, intensive care unit; VA/VV ECMO, veno–arterial/veno–venous extracorporeal membrane oxygenation*.

**Table 2:** Comparison of the characteristics of the survivors and non-survivors of VA-/VV-ECMO.

	**VA–ECMO survivors (*n =* 18)**	**VA–ECMO Non–survivors** **(*n =* 24)**	***P*– value**	**VV–ECMO survivors** **(*n =* 31)**	**VV–ECMO Non–survivors** **(*n =* 53)**	***P*– value**
Male, (*n*%)	7 (38.9)	13 (54.2)	0.327	15 (48.4)	24 (45.3)	0.783
Age (y)	36 ± 17	43 ± 14	0.132	39 ± 12	51 ± 13	**<0.001**
BMI (Kg/M^2^)	23.2 ± 4.2	23.0 ± 2.3	0.846	24.0 ± 3.9	23.9 ± 3.3	0.923
Remote shunt, (*n*%)	7 (38.9)	3 (12.5)	0.105	/	/	/
IABP, (*n*%)	12 (66.7)	14 (58.3)	0.582	/	/	/
CRRT, (*n*%)	11 (61.1)	16 (66.7)	0.710	23 (74.2)	32 (60.4)	0.199
SOFA score	10 (8–11)	12 (10.25–12.75)	**0.003**	8 (6–11)	11 (9.5–12)	**<0.001**
ACT pre–ECMO	143.72 ± 23.15	141.58 ± 21.04	0.756	150.71 ± 23.00	142.32 ± 20.32	0.086
ACT after–ECMO	394.39 ± 198.07	367.96 ± 182.75	0.657	371.74 ± 177.10	339.19 ± 149.34	0.371
ACT after–transported	214.78 ± 152.28	212.29 ± 98.41	0.949	220.39 ± 98.36	229.34 ± 149.39	0.767
APTT pre–ECMO	40.17 ± 6.30	40.32 ± 6.30	0.942	39.8 ± 12.55	40.57 ± 10.11	0.760
APTT after–transported	68.98 ± 36.50	79.21 ± 38.96	0.392	83.10 ± 42.79	85.46 ± 39.11	0.797
Sedation and analgesia						
Dexmedetomidine	18 (100)	24 (100)	–	31 (100)	53 (100)	–
Midazolam Maleate	13 (72.2)	19 (79.2)	0.720	10 (32.3)	23 (43.4)	0.361
Sufentanil	9 (50)	8 (33.3)	0.348	2 (6.5)	9 (17)	0.201
Remifentanil	9 (50)	16 (66.7)	0.348	29 (93.5)	44 (83)	0.201
Vasoactive agents						
Norepinephrine	10 (55.6)	12 (50)	0.764	1 (3.2)	4 (7.5)	0.647
Epinephrine	2 (11.1)	2 (8.3)	0.762	2 (6.5)	5 (9.4)	0.946
Dopamine	8 (44.4)	12 (50)	0.721	5 (16.1)	8 (15.1)	0.867
Dobutamine	4 (22.2)	5 (20.8)	0.914	1 (3.2)	1 (1.9)	0.998

*The data was shown as the mean ± SD, median (interquartile 25–75) or n (percentage). Bold values indicate statistical significance. BMI, body mass index; SOFA, sepsis–related organ failure assessment; IABP, intra–aortic balloon pump; CRRT, continuous renal replacement therapy; VA/VV ECMO, veno–arterial/veno–venous extracorporeal membrane oxygenation; ACT, activated clotting time; APTT, activated partial thromboplastin time*.

**Table 3:** Comparison of the complications of the survivors and non-survivors of VA-/VV-ECMO.

	**VA–ECMO survivors (*n =* 18)**	**VA–ECMO Non–survivors (*n =* 24)**	***P*– value**	**VV–ECMO survivors** **(*n =* 31)**	**VV–ECMO Non–survivors** **(*n =* 53)**	***P*– value**
Equipment complications, (*n*%)						
Clotting of ECMO system	1 (5.6)	1 (4.2)	0.973	2 (6.5)	3 (5.7)	0.846
Pump failure	0 (0)	1 (4.2)	0.755	0 (0)	0 (0)	–
ECMO system air embolism	0 (0)	0 (0)	–	1 (3.2)	1 (1.9)	0.998
Oxygen deficit	1 (5.6)	0 (0)	0.429	1 (3.2)	1 (1.9)	0.998
Patient complications, (*n*%)						
Respiratory and/or gastrointestinal tract hemorrhage	4 (22.2)	6 (25)	0.932	5 (16.1)	8 (15.1)	0.867
Intracranial hemorrhage	0 (0)	2 (8.3)	0.178	1 (3.2)	1 (1.9)	0.998
Bleeding on cannula site	13 (72.2)	14 (58.3)	0.517	8 (25.8)	24 (45.3)	0.104
Lower limb ischemia	1 (5.6)	2 (8.3)	0.676	/	/	/
Emergency orotracheal intubation	0 (0)	0 (0)	–	0 (0)	1 (1.9)	0.975
Cardiac arrest	0 (0)	1 (4.2)	0.755	0 (0)	0 (0)	–
Scald	1 (5.6)	0 (0)	0.429	0 (0)	0 (0)	–
Epilepsy	0 (0)	2 (8.3)	0.178	0 (0)	3 (5.7)	0.293

*The data was shown as n (percentage). VA/VV ECMO, veno–arterial/veno–venous extracorporeal membrane oxygenation*.

**Figure 2:**
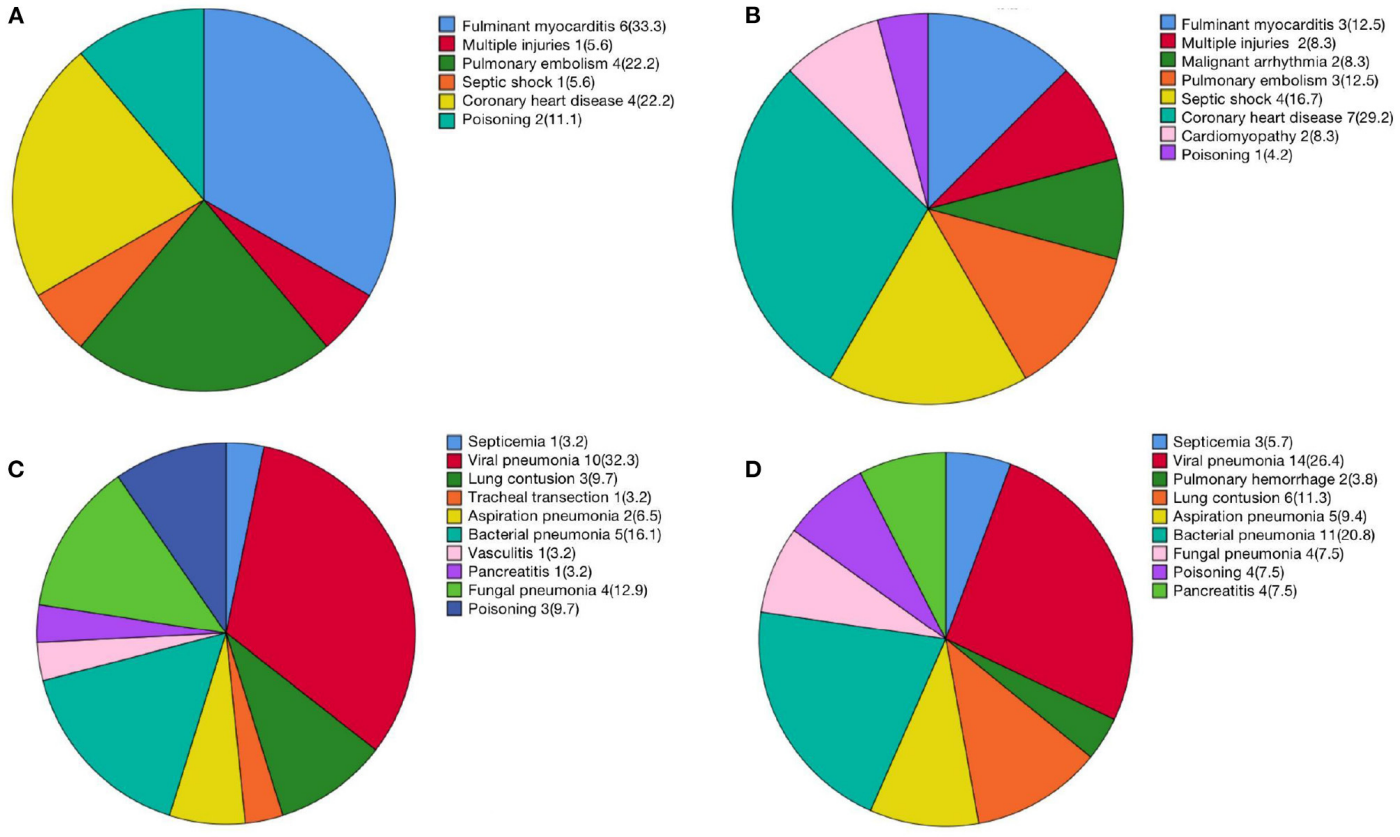
Numbers and percentages of ECMO indications. **(A)** VA-ECMO survivors; **(B)** VA-ECMO non-survivors; **(C)** VV-ECMO survivors; **(D)** VV-ECMO non-survivors.

Figure 2 described numbers and percentages of ECMO indications of different groups in detail. As shown in Figure 2; Table 1, the most common indication for VV-ECMO was pneumonia (*n* = 55, 43.7%), including viral pneumonia, bacterial pneumonia, fungal pneumonia, and aspiration pneumonia. Coronary heart disease (*n* = 11, 8.7%) and fulminant myocarditis (*n* = 9, 7.1%) were the important compositions of indications for VA-ECMO (Figure 1; Table 2).

Moreover, the levels of activated clotting time (ACT) and activated partial thromboplastin time (APTT) in the time of pre-transfer and post-tranfer, the percentage of sedation/ analgesia and vasoactive agents had no significant difference between survivors and non-survivors groups of VA-ECMO and VV-ECMO patients (all *p* > 0.05, Table 2).

### Description and Comparison of Complications During ECMO Transportation

Except for bleeding on cannula site, a total of 51 patients (40.5%) occurred complications during transportation, of which the percentage of patients' complications was 74.5% (Table 3). In detail, the VA-ECMO survivors were suffered 13 cases of bleeding on cannula site, 4 cases of respiratory and/or gastrointestinal tract hemorrhage, 1 case of lower limb ischemia, 1 case of scald, 1 case of clotting of ECMO system and 1 case of oxygen deficit during the transport. At the same time, the VA-ECMO non-survivors were appeared 13 cases of bleeding on cannula site, 6 cases of respiratory and/or gastrointestinal tract hemorrhage, 2 cases of intracranial hemorrhage, 2 cases of lower limb ischemia, 2 cases of epilepsy, 1 case of cardiac arrest, 1 case of clotting of ECMO system and 1 case of pump failure. Moreover, the transferred VV-ECMO survivors were suffered 8 cases of bleeding on cannula site, 5 cases of respiratory and/or gastrointestinal tract hemorrhage, 1 cases of intracranial hemorrhage, 2 cases of clotting of ECMO system, 1 case of ECMO system air embolism, and 1 case of oxygen deficit, while the VV-ECMO non-survivors were showed 24 cases of bleeding on cannula site, 8 cases of respiratory and/or gastrointestinal tract hemorrhage, 1 cases of intracranial hemorrhage, 3 cases of epilepsy, 1 case of emergency orotracheal intubation, 3 cases of clotting of ECMO system, 1 case of ECMO system air embolism, and 1 case of oxygen deficit (Table 3).

Among the patients who were occurred complications, the cases of hemorrhage was calculated to be the most common one (Table 3). As shown in Table 3, there was no significantly difference between the survivors and non-survivors of transferred VA and VV ECMO groups (all *p* > 0.05).

#### Comparison of the Monitoring Indicators Between the Survivors and Non-survivors of VA-/VV-ECMO in Different Time Points

**Figure 3:**
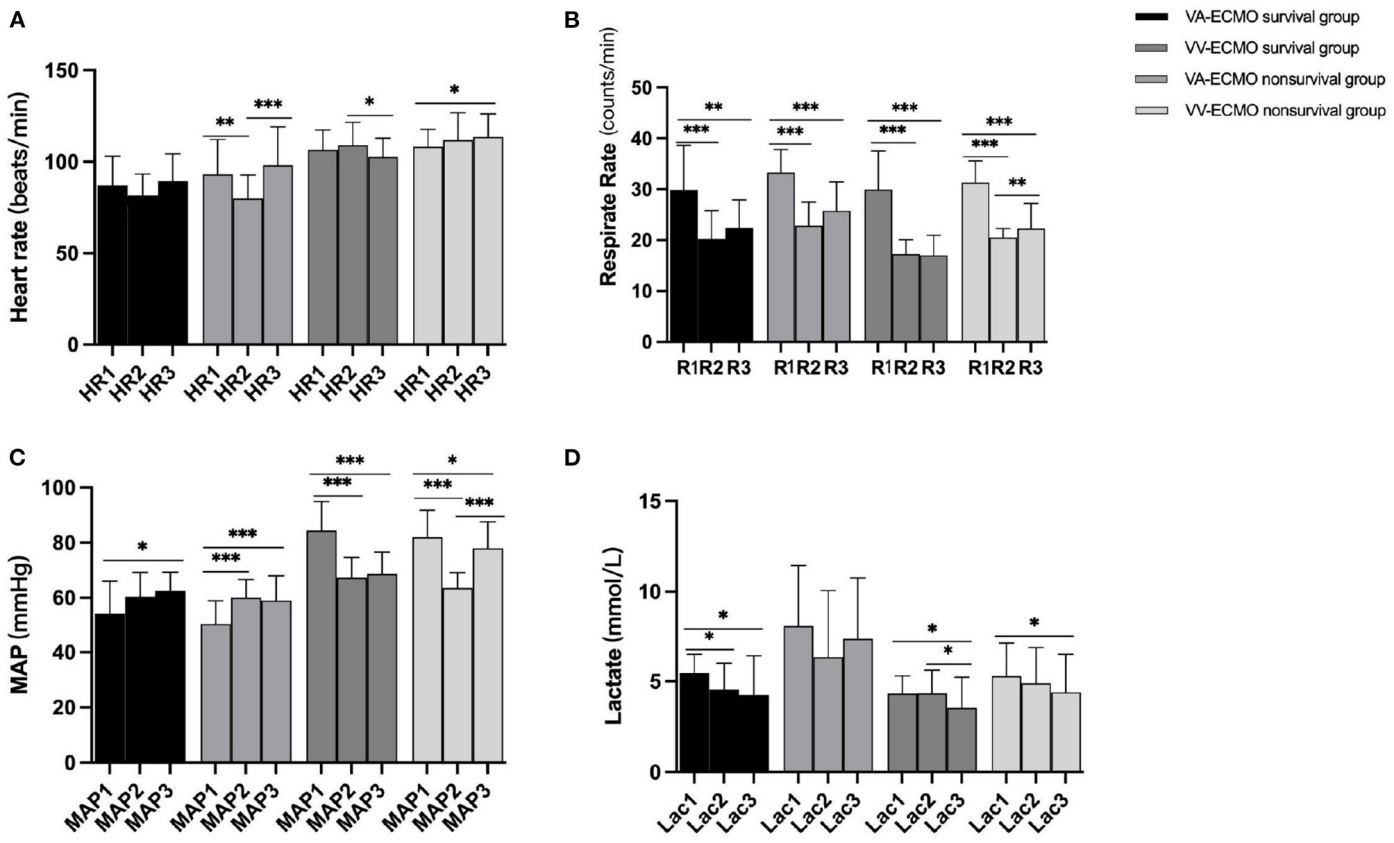
**(A–D)** Intra-group comparison of three time point (1= before ECMO boarding; 2= after ECMO boarding and patient's condition is relatively stable; 3= transported to the hospital). * indicate *P* < 0.05, **P < 0.01, and ***P < 0.001, respectively.

The monitoring indicators including HR, R, MAP, the levels of lactate, PaO_2_, and PaCO_2_ were recorded and analyzed. All of the monitoring indicators were obtained in three time points (before ECMO boarding, after ECMO boarding and patient's condition is relatively stable, transported to the hospital). After ECMO performed, the respiratory rate and the levels of lactate of the transferred ECMO patients were reduced and improved significantly (Figure 3). Interestingly, when compared the MAP of three different time points, the MAP of VA-ECMO patients was elevated while that of VV-ECMO patients was decreased (Figure 3).

Moreover, there was no significant difference in HR, R, MAP, PaO_2_, and PaCO_2_ between VA-ECMO survivors and non-survivors excepted the levels of lactate in three time points (before ECMO boarding: 5.45 ± 1.05 mmol/L vs. 8.11 ± 3.33 mmol/L, *P* < 0.001; after ECMO boarding and patient's condition is relatively stable: 4.57 ± 1.43 mmol/L 6.34 ± 3.72 mmol/L, *p* = 0.041; transported to the hospital: 4.26 ± 2.16 mmol/L vs. 7.36 ± 3.40 mmol/L, *P* < 0.001; respectively, Table 4). However, the survivors and non-survivors of the VV-ECMO manifested significantly differences in the baseline levels of lactate (4.33 ± 0.98 mmol/L vs. 5.29 ± 1.83mmol/L, *p* = 0.002, Table 4) and the VV-ECMO survivors seemed have lower HR and R but higher MAP after transportation (HR: 102.65 ± 10.34 beats/min vs. 113.51 ± 12.70 beats/min, *P* < 0.001; R: 17.00 ± 3.94 counts/min vs. 22.26 ± 4.98 counts/min, *P* < 0.001; MAP: 78 (72–86) mmHg vs. 62 (56.2–70.5) mmHg, *P* < 0.001; respectively, Table 4).

**Table 4:** Comparison of the indexes of the survivors and non-survivors of VA-/VV-ECMO.

	**VA–ECMO survivors (*n =* 18)**	**VA–ECMO Non–survivors** **(*n =* 24)**	***P*– value**	**VV–ECMO survivors** **(*n =* 31)**	**VV–ECMO Non–survivors** **(*n =* 53)**	***P*– value**
Heart rate (beats/min)						
Time point 1	87.00 ± 16.05	93.21 ± 19.02	0.259	106.55 ± 10.91	108.19 ± 9.65	0.491
Time point 2	81.50 ± 11.77	79.96 ± 12.90	0.689	109.06 ± 12.47	111.85 ± 14.94	0.362
Time point 3	89.44 ± 14.92	98.13 ± 20.89	0.124	102.65 ± 10.34	113.51 ± 12.70	**0.000**
Respiratory rate (counts/min)						
Time point 1	29.50 ± 8.75	33.33 ± 4.48	0.102	29.94 ± 7.58	31.30 ± 4.27	0.362
Time point 2	20.22 ± 5.59	22.83 ± 4.66	0.118	17.29 ± 2.76	20.51 ± 1.74	**0.000**
Time point 3	22.44 ± 5.51	25.75 ± 5.67	0.065	17.00 ± 3.94	22.26 ± 4.98	**0.000**
Mean arterial pressure (MAP) (mmHg)						
Time point 1	82 (78–90)	78 (72.5–88.25)	0.239	85 (78–90)	54 (46.5–69.5)	**0.018**
Time point 2	65.5 (63–75)	64 (60–66)	0.884	64 (61–67)	60 (56–64)	0.294
Time point 3	66.5 (62.25–75)	67.5 (64–74.25)	0.155	78 (72–86)	62 (56.2–70.5)	**0.000**
Lactate level (mmol/L)						
Time point 1	5.45 ± 1.05	8.11 ± 3.33	**0.000**	4.33 ± 0.98	5.29 ± 1.83	**0.002**
Time point 2	4.57 ± 1.43	6.34 ± 3.72	**0.041**	4.43 ± 1.28	4.89 ± 1.98	0.123
Time point 3	4.26 ± 2.16	7.36 ± 3.40	**0.000**	3.55 ± 1.68	4.40 ± 2.11	**0.046**
Arterial partial pressure of oxygen (PaO_2_) (mmHg)						
Time point 1	60.22 ± 15.07	54.63 ± 11.84	0.202	49.25 ± 10.87	51.44 ± 5.44	0.300
Time point 2	144.33 ± 70.24	174.83 ± 96.30	0.242	79.44 ± 15.51	81.03 ± 13.67	0.637
Time point 3	157.78 ± 60.47	173.46 ± 64.95	0.426	83.26 ± 8.77	86.19 ± 11.79	0.198
Arterial partial pressure of carbon dioxide (PaCO_2_) (mmHg)						
Time point 1	39.78 ± 10.07	34.25 ± 12.09	0.114	61.88 ± 18.65	57.55 ± 15.02	0.276
Time point 2	30.50 ± 7.91	33.21 ± 6.33	0.241	39.28 ± 9.74	35.88 ± 9.81	0.128
Time point 3	29.17 ± 3.63	30.25 ± 6.10	0.478	34.57 ± 11.01	30.30 ± 8.92	0.072

*The data was shown as the mean ± SD or median (interquartile 25–75). Bold values indicate statistical significance. VA/VV ECMO, veno–arterial/veno–venous extracorporeal membrane oxygenation. Time point 1, before ECMO boarding; Time point 2, after ECMO boarding and patient's condition is relatively stable; Time point 3, transported to the hospital*.

#### Comparison of the Outcomes Between the Survivors and Non-survivors of VA-/VV-ECMO

Age, SOFA score, MAP after transportation, and the baseline levels of lactate were finally choose as variables for the Cox regression survival analysis of VA-ECMO and VV-ECMO transferred patients. As shown in Table 5, the high SOFA scores and the baseline lactate levels were indicated the poor prognosis of transferred VA-ECMO patients (SOFA score: 1.562 (1.135–2.151), *p* = 0.006; Lactate level: 1.135 (1.015–1.268), *p* = 0.026; respectively). Concerning to the outcomes of VV-ECMO transferred patients, older age and lower MAP after transportation were associated the high ICU mortality excepted the high SOFA scores and the baseline lactate levels (age: 1.022 (1.001–1.044), *p* = 0.045; MAP after transportation: 0.939 (0.914–0.965), *P* < 0.001; SOFA score: 1.200 (1.054–1.366), *p* = 0.006; Lactate level: 1.285 (1.084–1.522), *p* = 0.004; respectively, Table 5).

**Table 5:** Cox regression survival analysis of VA–ECMO and VV–ECMO patients.

	**VA–ECMO patients**	***P*–value**	**VV–ECMO patients**	***P*–value**
Age	–	0.764	1.022(1.001–1.044)	**0.045**
SOFA score	1.562 (1.135–2.151)	**0.006**	1.200 (1.054–1.366)	**0.006**
MAP after transportation	–	0.488	0.939 (0.914–0.965)	**<0.001**
Lactate level	1.135 (1.015–1.268)	**0.026**	1.285 (1.084–1.522)	**0.004**

*The data was shown as the Exp (B) and 95%CI. Bold values indicate statistical significance. VA/VV ECMO, veno–arterial/veno–venous extracorporeal membrane oxygenation; SOFA, sepsis–related organ failure assessment; MAP, mean arterial pressure*.

**Figure 4:**
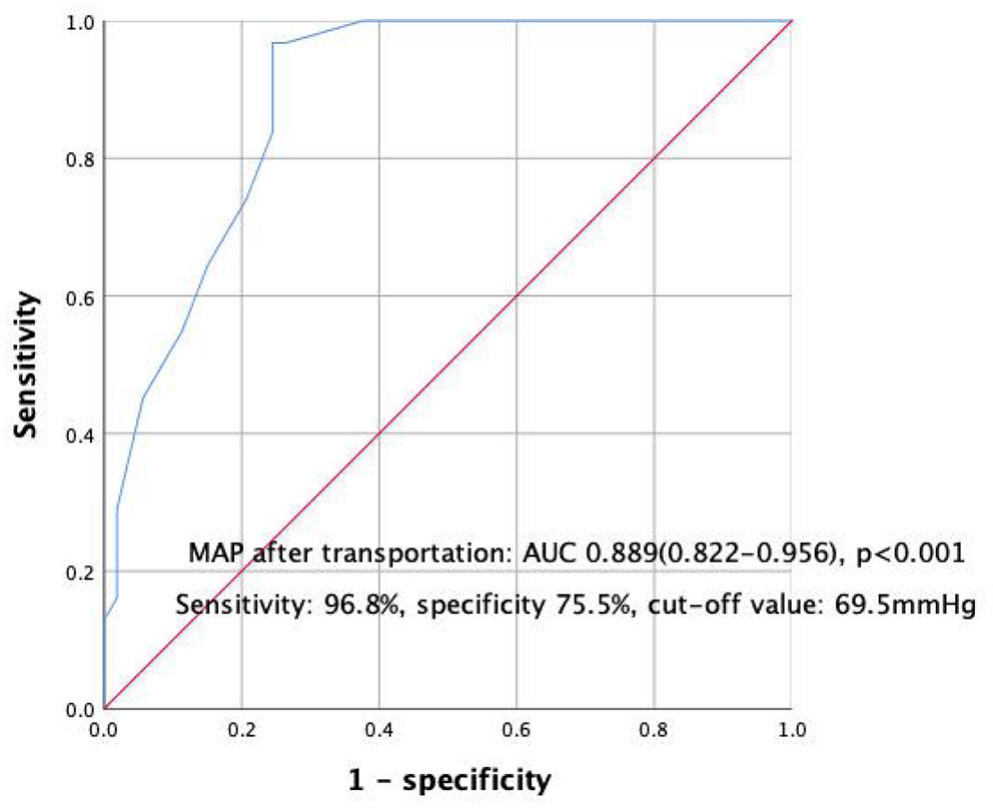
ROC curve of VV-ECMO patients' MAP (mean arterial pressure).

**Figure 5:**
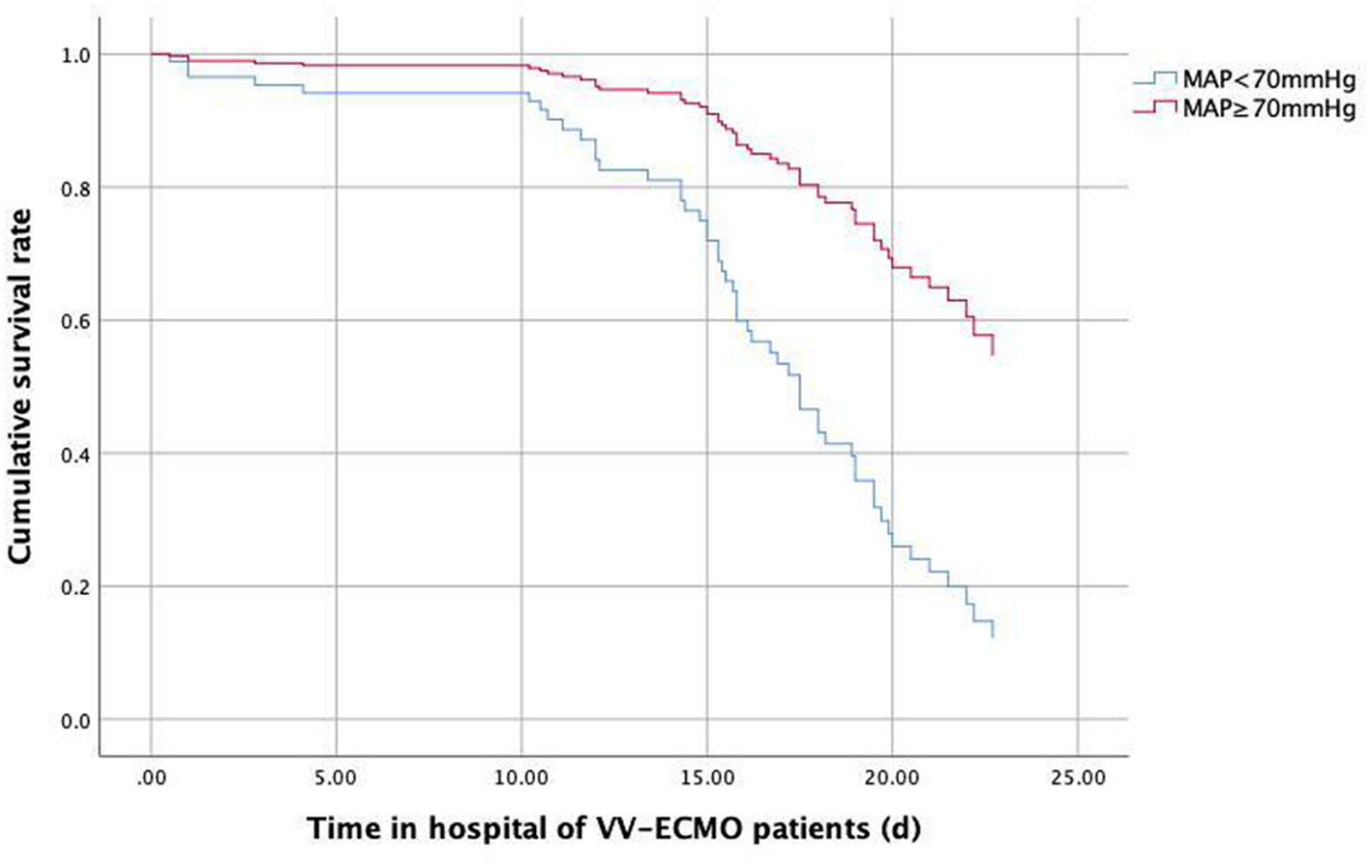
Cox regression survival analysis curve of VV-ECMO patients' MAP.

The AUC of lower MAP after transportation for the mortality of transferred VV-ECMO patients were 0.889 (95%CI:0.822–0.956, *P* < 0.001, Figure 4) and the cut-off value of MAP after transportation was 69.5 mmHg with a high sensitivity and specificity (sensitivity: 96.8%, specificity 75.5%, Figure 4). Then, the Cox regression survival analysis curve of VV-ECMO patients' MAP showed that the survival rate could improved when the MAP after transportation not <70 mmHg (Figure 5).

## Discussion

The present study retrospectively analyzed 84 VV-ECMO and 42 VA-ECMO critically ill adults patients primary transferred by our mobile ECMO team and all of the cannulation is performed through percutaneously by ECMO physician. The ECMO transport is feasible as no death occurred in our study. The results of our study showed that the higher baseline lactate and SOFA score indicated poor prognosis of all ECMO transport patients, and lower MAP after transportation (<70 mmHg) had potential predictive value for short-term outcome of VV-ECMO patients.

In recent years, ECMO has played an increasingly important role in saving critically ill patients. However, the regional hospitals and the relatively small-volume centers are often inadequately equipped and inexperienced, which increases patient mortality. After the ELSO has collected a large number of ECMO patients and ECMO transport data, it is recommended that critical patients be transported to a large ECMO center for further treatment after evaluation, which may improve the survival rate of critically ill patients (1). To date, there have been many published studies on ECMO transport, most of which only included patients who were transported by VV-ECMO, and included some neonates and infants. In addition, there are no recognized guidelines for ECMO transfer, such as the composition of the ECMO transfer team, the indicators that need to be paid attention to during the transportation, and the occurrence and treatment of complications during the transfer. Therefore, the characteristics of this study are that all the included patients are adults, and the possible risk factors that affect the prognosis of VA-ECMO and VV-ECMO transport patients are separately discussed.

Our mobile ECMO team consists of ECMO physician, cardiac surgeon, and intensive care nurse, and they are experienced in prehospital emergency medicine, ECMO physiology, ECMO technology, and intensive care. The ECMO physician will handled all transport-related coordination and performed percutaneously vessels' cannulation. The mobile ECMO team was in charge of the ECMO circuit, the ventilator, medications, the application of heparin, and resolve complications of transferred critically ill patient. The feasibility of inter-hospital ECMO transfer is supported by most published data, and during transport, the mortality rate is reported low than 0.5% (11). In a recent literature review, authors analyzed 2,647 transferred patients reported in the years 2013 to 2019, and found that there were 4 deaths (mortality rate: 0.15%) were directly associated to medical transfers (6–9). Our ECMO team was experienced and no death occurred during the transportation for ECMO patients in our center till now.

However, life-threatening complications might occur during ECMO transport. The complications or adverse events during transport often be categorized into five major groups, including patient, equipment, vehicle, environment and personnel. In the Karolinska center papers published by Broman et al., the complications' percentage distribution of 322, 514, and 908 transfers were described in detail, and then reported that complications were predominantly patient-related (70, 65, and 62%, respectively) (8, 12, 13). In our center, the percentage of patients complications was 74.5% (Table 3), and hemorrhage was occupied the most common complication. The high incidence of hemorrhage is due to the application of anticoagulant drugs, blood vessel damage caused by intubation, and patient movement after intubation.

In 2019, Dalia et al. analyzed the data of 51 transferred and 215 in-house ECMO supported patients, and the survival rate of the two groups showed no significant difference (5). Lactate and SOFA scores are currently recognized predictors of the poor prognosis of ECMO supported patients. Recently, a observation in the Danish population revealed that the lower levels of lactate was associated to higher survival rate with a good neurological outcome (14). A recent study included 106 patients revealed that the progressive hyperlactatemia after VA-ECMO initiation for adult patients with cardiogenic shock is a sensitive and specific predictor of hospital mortality (15). Another study with 72 patients with cardiac arrest demonstrated that the metabolic state, expressed as level of lactate just before VA-ECMO initiation seems more predictive of outcome than cardiopulmonary resuscitation duration or absence of return of spontaneous circulation (16). Results from a real-world clinical experience with the percutaneous extracorporeal life support system suggested that only serum lactate concentration at admission could be proven as independent predictor of patients' outcome, and patients with lactate concentrations above 10 mmol/L exhibited > 95% mortality (17). Wu et al. found that SOFA score calculated before ECMO showed the prognostic value in a cohort of 45 patients treated with ECMO for cardiac or respiratory failure (18). Lindskov et al. showed that the SOFA score calculated at day 1 after ECMO initiation was a predictive factor of low survival rate (19). Roch et al. demonstrated that SOFA was associated with mortality prior to ECMO in ARDS patients treated with VV-ECMO who have all been cannulated in distant hospitals (20). Our present study also believed that the baseline lactate and SOFA scores before ECMO preformed were related to the prognosis of transferred ECMO patients.

However, few published literature reported whether changes in monitoring indicators (MAP, HR, et al.) during patient transfer have an impact on the ECMO supported transport patients' prognosis. Sun et al. reported that average MAP <65 mmHg in the first 6 h of ECPR indicates a poor neurological prognosis for ECPR patients (21). Our study retrospectively enrolled 126 adult VA and VV ECMO patients transferred from regional hospital to the First Affiliated Hospital of Zhengzhou University by our ECMO team during June 2012 to Sept 2020, and found that lower MAP after transportation (<70 mmHg) had potential predictive value for short-term outcome of VV-ECMO patients, while the SOFA score and the lactate level could be used to evaluate the risk of ICU mortality of transportation ECMO patients.

This study is a single-center retrospective study with a small sample size, so the conclusion still needs a multi-center, large-sample, prospective randomized controlled study to further verify. Moreover, the present study only enrolled transferred ECMO patients, but it not included in-hospital ECMO patients. There might existed different mortality predictors between the transport and in-hospital ECMO patients. In addition, our study selected indicators at different time points to analyzing. Perhaps it is more meaningful to choose the average value of the special time periods for find the valuable outcome predictors of transferred VA and VV ECMO patients. Finally, the vehicle of transport need to be further improved, for example, long-distance transportation can use helicopters.

## Conclusion

The transport of ECMO supported patients by our experienced mobile ECMO team is feasible although life-threatening complications might occurred during transportation. The baseline lactate and SOFA score are predictors of transportation ECMO patients' ICU mortality. Besides, lower MAP after transportation (<70 mmHg) had potential predictive value for poor prognosis of transferred VV-ECMO patients but not VA-ECMO ones.

## Data Availability Statement

The raw data supporting the conclusions of this article will be made available by the authors, without undue reservation.

## Ethics Statement

The studies involving human participants were reviewed and approved by the Ethics Committee of the First Affiliated Hospital of Zhengzhou University (No. 2020-KY-429). The patients/participants provided their written informed consent to participate in this study. Written informed consent was obtained from the individual(s) for the publication of any potentially identifiable images or data included in this article.

## Author Contributions

Y-CZ and XZ designed the study and wrote the first draft of the manuscript. JL, G-WF, and M-JH verified data extraction, data analysis, and reviewed the manuscript. HZ, X-XL, and Z-QW supervised the data acquisition, data analysis, and interpretation. All authors read and approved the final manuscript.

## Funding

This study was partly supported by Joint Project of Medical Science and Technology of Henan (LHGJ20190095) awarded to XZ.

## Conflict of Interest

The authors declare that the research was conducted in the absence of any commercial or financial relationships that could be construed as a potential conflict of interest.

## Publisher's Note

All claims expressed in this article are solely those of the authors and do not necessarily represent those of their affiliated organizations, or those of the publisher, the editors and the reviewers. Any product that may be evaluated in this article, or claim that may be made by its manufacturer, is not guaranteed or endorsed by the publisher.

## References

[1] Extracorporeal Life Support Organization. Guidelines for ECMO Transport Ann Arbor, ELSO, Ann Arbor, MI, USA. (2015). Available online at: https://www.elso.org/Portals/0/Files/ELSO%20GUIDELINES%20FOR%20ECMO%20TRANSPORT_May2015.pdf (accessed February 2018).

[2] Lindén V Palmér K Reinhard J Westman R Ehrén H Granholm T. Inter-hospital transportation of patients with severe acute respiratory failure on extracorporeal membrane oxygenation-national and international experience. Intensive Care Med. (2001) 27:1643–8. 10.1007/s001340101060 11685306

[3] Starck C Hasenclever P Falk V Wilhelm MJ. Interhospital transfer of seriously sick ARDS patients using veno-venous extracorporeal membrane oxygenation (ECMO): concept of an ECMO transport team. Int J Crit Illn Inj Sci. (2013) 3:46–50. 10.4103/2229-5151.109420 23724385 PMC3665119

[4] Wiegersma JS Droogh JM Zijlstra JG Fokkema J Ligtenberg JJ. Quality of interhospital transport of the critically ill: impact of a mobile intensive care unit with a specialized retrieval team. Crit Care. (2011) 15:R75. 10.1186/cc10064 21356054 PMC3222008

[5] Dalia AA Axtel A Villavicencio M D'Allesandro D Shelton K Cudemus G. A 266 Patient experience of a quaternary care referral center for extracorporeal membrane oxygenation with assessment of outcomes for transferred vs. in-house patients. J Cardiothorac Vasc Anesth. (2019) 33:3048–53. 10.1053/j.jvca.2019.05.017 31230966

[6] Bryner B Cooley E Copenhaver W Brierley K Teman N Landis D. Two decades' experience with interfacility transport on extracorporeal membrane oxygenation. Ann Thorac Surg. (2014) 98:1363–70. 10.1016/j.athoracsur.2014.06.025 25149055

[7] Bréchot N Mastroianni C Schmidt M Santi F Lebreton G Hoareau AM. Retrieval of severe acute respiratory failure patients on extracorporeal membrane oxygenation: Any impact on their outcomes? J Thorac Cardiovasc Surg. (2018) 155:1621–9. 10.1016/j.jtcvs.2017.10.084 29246547

[8] Fletcher-Sandersjöö A Frenckner B Broman M. A single-center experience of 900 interhospital transports on extracorporeal membrane oxygenation. Ann Thorac Surg. (2019) 107:119–27. 10.1016/j.athoracsur.2018.07.040 30240763

[9] Puslecki M Baumgart K Ligowski M Dabrowski M Stefaniak S Ladzinska M. Patient safety during ECMO transportation: single center experience and literature review. Emerg Med Int. (2021) 2021: 6633208. 10.1155/2021/6633208 33688436 PMC7920709

[10] Organization ECLS ELSO. Anticoagulation guideline the extracorporeal life support organization (ELSO). Ann Arbor. MI, USA. (2014) Available online at: https://www.elso.org/ecmo-resourcws/elso-ecmo-guidelines.aspx. (accessed April 2018).

[11] Barbaro RP Odetola FO Kidwell KM Paden ML Bartlett RH Davis MM. Association of hospital-level volume of extracorporeal membrane oxygenation cases and mortality. analysis of the extracorporeal life support organization registry. Am J Respir Crit Care Med. (2015) 191:894–901. 10.1164/rccm.201409-1634OC 25695688 PMC4435456

[12] Broman LM Frenckner B. Transportation of critically ill patients on extracorporeal membrane oxygenation. Front Pediatr. (2016) 4:63. 10.3389/fped.2016.00063 27379221 PMC4904149

[13] Ericsson A Frenckner B Broman LM. Adverse events during inter-hospital transports on extracorporeal membrane oxygenation. Prehosp Emerg Care. (2017) 21:448–55. 10.1080/10903127.2017.1282561 28166435

[14] Mørk SR Stengaard C Linde L Møller JE Jensen LO Schmidt H. Mechanical circulatory support for refractory out-of-hospital cardiac arrest: a danish nationwide multicenter study. Crit Care. (2021) 25:174. 10.1186/s13054-021-03606-5 34022934 PMC8141159

[15] Laimoud M Alanazi M. The clinical significance of blood lactate levels in evaluation of adult patients with veno-arterial extracorporeal membrane oxygenation. Egypt Heart J. (2020) 72:74. 10.1186/s43044-020-00108-7 33108534 PMC7588953

[16] Fux T Holm M Corbascio M van der Linden J. Cardiac arrest prior to venoarterial extracorporeal membrane oxygenation: risk factors for mortality. Crit Care Med. (2019) 47:926–33. 10.1097/CCM.0000000000003772 31094743

[17] Masyuk M Abel P Hug M Wernly B Haneya A Sack S. Real-world clinical experience with the percutaneous extracorporeal life support system: results from the german lifebridge registry. Clin Res Cardiol. (2020) 109:46–53. 10.1007/s00392-019-01482-2 31028475

[18] Wu MY Lin PJ Tsai FC Haung YK Liu KS Tsai FC. Impact of preexisting organ dysfunction on extracorporeal life support for nonpostcardiotomy cardiopulmonary failure. Resuscitation. (2008) 79:54–60. 10.1016/j.resuscitation.2008.05.002 18617313

[19] Lindskov C Jensen RH Sprogoe P Klaaborg KE Kirkegaard H Severinsen IK. Extracorporeal membrane oxygenation in adult patients with severe acute respiratory failure. Acta Anaesthesiol Scand. (2013) 57:303–11. 10.1111/aas.12050 23278552

[20] Roch A Hraiech S Masson E Grisoli D Forel JM Boucekine M. Outcome of acute respiratory distress syndrome patients treated with extracorporeal membrane oxygenation and brought to a referral center. Intensive Care Med. (2014) 40:74–83. 10.1007/s00134-013-3135-1 24170143 PMC7095017

[21] Sun F Mei Y Lv JR Li W Hu D Zhang G. Average mean arterial pressure in the first 6 hours of extracorporeal cardiopulmonary resuscitation in the prediction of the prognosis of neurological outcome: a single-center retrospective study. Perfusion. (2021) 1:e027118 10.1177/02676591211027118 34213369

